# Immediate, short-term, and long-term effects of balloon mitral valvuloplasty on the left atrial global longitudinal strain and its correlation to the outcomes in patients with severe rheumatic mitral stenosis

**DOI:** 10.1186/s43044-023-00425-7

**Published:** 2023-12-01

**Authors:** Amr Setouhi, Hesham Boshra, Hany Askalany, Hazem M. A. Farrag

**Affiliations:** 1 Cardiology Department, Faculty of Medicine, Minia University, Minia, Egypt; 2 Cardiology Department, Faculty of Medicine, Beni Suef University, Beni Suef, Egypt

**Keywords:** Balloon mitral valvuloplasty, Speckle tracking echocardiography, BMV, Global longitudinal left atrial strain

## Abstract

**Background:**

Left atrial global longitudinal strain (LA GLS) has been used as a new assessment tool for left atrial function. This article aims to investigate the effect of balloon mitral valvuloplasty (BMV) in patients with severe rheumatic mitral stenosis on LA GLS and its relation to the mitral valve area achieved after the procedure. The study included 95 patients with severe mitral stenosis who fulfilled the criteria for BMV (case group) and 80 normal healthy subjects (control group). All included participants underwent complete echocardiographic examinations. For the case group, LA GLS was assessed by 2D speckle-tracking Echocardiography before valvuloplasty, immediately after, within 24 h, at 6 months, and at 12 months, and the results were compared.

**Results:**

The impaired left-atrium strain in patients with severe mitral stenosis was improved immediately after BMV, and the improvement continued at 6 and 12 months post-BMV (23.1% ± 4.2 vs. 36.0% ± 4.9, 36.2% ± 4.5, and 40.1% ± 9.5, respectively *p* < 0.01). After BMV, there was a significant decrease in left atrial volume (76.3 ± 12.4 ml/m^2^ vs. 68.6 ± 10.4 ml/m^2^, *p* < 0.01) and a significant increase in the area occupied by the mitral valve (1.02 ± 0.18 cm^2^ vs. 1.60 ± 0.31 cm, *p* < 0.01). The immediate LA GLS and the mitral valve area were positively correlated (*r* = 0.64, *p* < 0.01). Furthermore, the immediate LA GLS was associated with significantly improved function class (*p* < 0.01).

**Conclusions:**

LA GLS can indicate left atrial (mainly reservoir) function. The improvement observed in patients after BMV may indicate that LA GLS can be used to evaluate the progress after BMV.

*Trial registration:* The study was approved by the local ethics committee of the Faculty of Medicine in Minia University (Registration No. MUFMIRB 324-4-2022). Institutional Review Board, Faculty of Medicine, Minia University, Egypt. 324-4-2022, 24 18 April, 2022.

## Background

The incidence of rheumatic heart disease has recently decreased, but the disease still has very high morbidity and mortality rates, which are particularly prevalent in underdeveloped countries [1]. The preferred treatment for severe rheumatic mitral stenosis (MS) is percutaneous balloon mitral valvuloplasty (PBMV) [2, 3]. This method has advantages of decreasing mortality rates and hospital stays compared to conventional mitral valve surgery [3]. Many studies have reported favorable immediate and short-term outcomes, but long-term follow-up data are insufficient and need more assessment [4, 5].

During rheumatic MS, valve narrowing occurs over years, which is why the condition is considered a progressive disease [3]. Various mechanisms have been proposed to describe left ventricle (LV) systolic dysfunction in MS patients. These mechanisms include chronic reduction in preload, which can contribute to adverse LV remodeling. The inflammatory process may also extend from the mitral valve apparatus into the surrounding myocardium [6]. MS is classified according to the mitral valve area (MVA). Moderate to severe MS is considered when the MVA is less than or equal to 1.5 cm^2^, while mild MS is considered when the MVA exceeds 1.5 cm^2^ [7].

Kanji Inoue first described BMV in 1982 [8]. Before this percutaneous method was developed, most symptomatic MS patients were treated using two methods: closed and open surgical commissurotomy. BMV has demonstrated similar or sometimes greater success rates and comparable restenosis rates in comparison with surgical commissurotomy [9]. Two-dimensional speckle-tracking Echocardiography can efficiently quantify left atrial global longitudinal strain (LA GLS) and is emerging as a novel tool for assessing left atrial function [5].

The objective of this study was to examine the impact of BMV in patients with severe rheumatic MS on LA GLS and its relation to the MVA and examine the outcome observed after the procedure.

## Methods

### Study settings and population

This prospective case–control study included 95 patients who had severe long-standing MS and fulfilled the criteria for BMV, as well as 80 healthy control subjects. Patients diagnosed with MVA less than 1.5 cm^2^ who suffered from severe MS were included in the study. Patients were excluded if they had aortic stenosis, mitral or aortic regurgitation, atrioventricular conduction anomalies, atrial fibrillation, ischemic heart diseases, chronic kidney diseases, heart failure, chronic obstructive pulmonary disease, heart disease, cardiomyopathies, previous percutaneous balloon valvuloplasty, hypertension, diabetes mellitus, anemia, or systemic disease. Informed consent was obtained from all participants.

A detailed history was obtained from the included cases, clinical inspection was performed, and electrocardiograms (ECGs) were recorded. Transthoracic 2D Echocardiography was performed on all patients before and after valvuloplasty and included 2D echo, continuous wave, and colored flow-mapping Doppler measurements. Standard views were obtained using a Philips CX50, IE 33 X Matrix, and GE Vivid 5 with S3 and X5-1 matrix array transducers and harmonic imaging. The patient was positioned in a left lateral position with continuous ECG monitoring.

The examination was executed in accordance with the recommendations of the American Society of Echocardiography [10]. The data collected during imaging and examinations were stored in a workstation for offline analysis. A senior echocardiographer with more than 10 years of experience performed echocardiographic examinations and follow-up measurements.

### BMV

BMV was performed using an antegrade trans-septal approach with a Multitrack balloon or Inoue balloon for all patients. After the procedure, a post-procedural hemodynamic assessment was performed to evaluate the trans-mitral pressure gradients and left atrial pressure.

### Follow-up

Two-dimensional speckle-tracking Echocardiography was used to examine LA GLS before performing the valvuloplasty, and patients who had a successful valvuloplasty were assessed immediately after the procedure, within 24 h, at 6 months, and 12 months.

### Statistical analysis

The data analysis was performed using the statistical analysis software SPSS (version 20, IBM, NY, USA) [11]. The mean ± standard deviation (SD) was used to express the continuous variables. A chi-squared test was employed to examine categorical data. An independent t-test was used to estimate the differences between groups. Variations between before and after BMV in the case group were compared using paired t-tests. The significance of correlations between two variables was investigated using the Pearson correlation coefficient. The criterion for statistical significance was a *p*-value of < 0.05.

## Results

Table 1 presents the demographic and baseline data of the study groups. The case group comprised 95 individuals, while the control group comprised 80. In the case group, there were 71 female patients (74.7%) and 24 male patients (25.2%), and the mean age was 29 ± 9.2 years. Both groups were comparable regarding the body mass index and surface area, as shown in Table 1.

**Table 1:** Demographic and baseline data of all the studied groups

Variable	Cases (n = 95)	Cont (n = 80)	*p* value (Sig.)
Age (year)	29.1 ± 9.2	28.4 ± 8.2	0.60^NS^
Sex (Male/female)	24/71	29/66	0.65^NS^
Body mass index (kg/m^2^)	22.9 ± 3.8	22.1 ± 4.8	0.22^NS^
Body surface area (m^2^)	2.08 ± 0.14	2.04 ± 0.19	0.10^NS^
SBP (mmhg)	115.2 ± 4.7	118.2 ± 4.1	< 0.01**
DBP (mmhg)	74.9 ± 3.8	78.8 ± 4.0	< 0.01**
LA (IS in mm)	62.7 ± 8.4	43.2 ± 7.8	< 0.01**
LA area (in cm^2^)	27.5 ± 7.4	12.4 ± 4.8	< 0.01**
LA strain (%)	12.9 ± 1.2	31.4 ± 4.7	< 0.01**

*NS* Non-Significant

**:* P*-value > 0.05: Non-significant;* P*-value < 0.05: Significant;* P*-value < 0.01: highly significant

Table 2 presents the echocardiography parameters of the case and control groups. In regard to echocardiographic parameters, the case group had significantly lower LA GLS (23.1% ± 4.2), lower MVA (1.02 ± 0.18 cm^2^), higher sPAP (43.7 ± 9.1 mm Hg), and higher LAV index (76.3 ± 12.4 ml/m^2^) compared to the control group (40.3% ± 5.9, 4.58 ± 0.82 cm^2^, 21.5 ± 6.8 mm Hg, and 68.6 ± 10.4 ml/m^2^, respectively). Table 3 shows the changes in echocardiogram parameters before and after valvuloplasty in the case group. After valvuloplasty, there was significantly higher LA GLS (34.7% ± 4.9), larger MVA (1.6 ± 0.3 cm^2^), lower sPAP (12.6 ± 6.4 mm Hg), and lower LAV index (64.9 ± 14.7 ml/m^2^) in comparison with values from before the procedure (23.1% ± 4.2, 1.02 ± 0.18 cm^2^, 43.7 ± 9.1 mm Hg, and 76.3 ± 12.4 ml/m^2^, respectively).

**Table 2:** Echocardiography parameters between cases and control group

Variable	Cases (n = 95)	Control (n = 80)	*p* value (Sig.)
LA GLS(%)	23.1 ± 4.2	40.3 ± 5.9	< 0.01**
MVA(in cm^2^)	1.02 ± 0.18	4.58 ± 0.82	< 0.01**
SPAP(mm Hg)	43.7 ± 9.1	21.5 ± 6.8	< 0.01**
LAV index (mL/m^2^)	76.3 ± 12.4	68.6 ± 10.4	< 0.01**

*LAV*: left atrial volume index, *SPAP*: systolic pulmonary arterial pressure, and *MVA*: mitral valve area

**:* P*-value > 0.05: Non-significant;* P*-value < 0.05: Significant;* P*-value < 0.01: highly significant

**Table 3:** Difference in echocardiogram parameters before and after valvuloplasty in cases group

Variable	Pre-procedural	Post-procedural	*p* value (Sig.)
LA GLS(%)	23.1 ± 4.2	34.7 ± 4.9	< 0.01**
MVA(in cm^2^)	1.02 ± 0.18	1.60 ± 0.31	< 0.01**
SPAP(mm Hg)	43.7 ± 9.1	12.6 ± 6.4	< 0.01**
LAV index (mL/m^2^)	76.3 ± 12.4	64.9 ± 14.7	< 0.01**

**:* P*-value > 0.05: Non-significant;* P*-value < 0.05: Significant;* P*-value < 0.01: highly significant

**Fig. 1:**
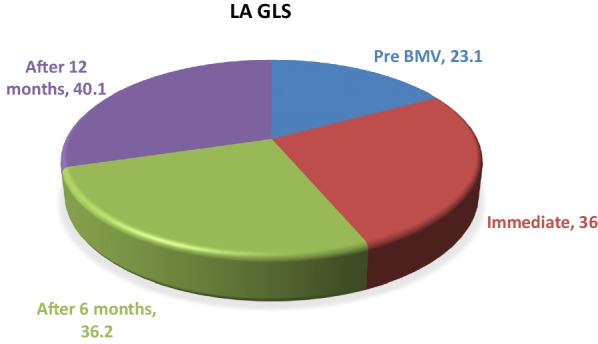
Left atrial global longitudinal strain follow-up before and after mitral balloon valvuloplasty

Table 4 shows information related to the function class and pulmonary artery pressure from before and after valvuloplasty in the case group to illustrate the impact of the valvuloplasty procedure. The function class was improved significantly after valvuloplasty (*p* < 0.01), as shown in Table 4. Table 5 presents the results of the LA GLS from before and after the BMW procedure. LA GLS was worse in patients with severe MS, but it improved immediately after BMV, at 6 months, and at 12 months (23.1 ± 4.2 vs. 36.0 ± 4.9, 36.2 ± 4.5, and 40.1 ± 9.5, *p* < 0.01), as shown in Table 5 and Fig. 1. The results indicated a strong, positive, significant correlation between immediate LA GLS and the achieved MVA (r = 0.64, *p* < 0.01). Table 6 illustrates the correlation between immediate LA GLS and MVA after BMW, which indicates that the immediate LA GLS was associated with a significant improvement in function class (*p* < 0.01).

**Table 4:** Function class and pulmonary artery pressure before and after valvuloplasty in cases group

		Pre-procedural	Post-procedural	*p* value (Sig.)
Function class	Class (I)	0	52 (52.0%)	< 0.01**
	Class (II)	19 (19.0%)	41 (41.0%)	
	Class (III)	48 (48.0%)	7 (7.0%)	
	Class (IV)	33 (33.0%)	0	
Pulmonary artery	Pressure	47.4 ± 7.9	36.3 ± 6.5	< 0.01**

T-test and Chi-square test were used; p < 0.01(significant)

**:* P*-value > 0.05: Non-significant;* P*-value < 0.05: Significant;* P*-value < 0.01: highly significant

**Table 5:** Left atrial global longitudinal strain follow-up before and after BMW

Variable	LA GLS				
	Pre BMV	Immediate	After 6 months	After 12 months	*p* value (Sig.)
Mean (%)	23.1^c^	36.0 ^b^	36.2 ^b^	40.1^a^	< 0.01**
SD (%)	4.2	4.9	4.5	9.5	< 0.01**

^a,b,c^Means with different superscript letters are significantly different

Repeated measures ANOVA test to compare LA GLS revealed; p < 0.01(significant)

BMV: mitral balloon valvuloplasty, SD: standard deviation

**:* P*-value > 0.05: Non-significant;* P*-value < 0.05: Significant;* P*-value < 0.01: highly significant

**Table 6:** Correlation between LA GLS immediately post BMW and MVA

Variable	Correlation coefficient (r)	*p* value (Sig.)
Correlation between Immediate LA GLS and MVA	0.64	< 0.01**
Correlation between Immediate LA GLS and PAP	− 0.62	< 0.01**
Correlation between Immediate LA GLS and function class	− 0.59	< 0.01**

Person and Spearman correlation coefficients were used

**:* P*-value > 0.05: Non-significant;* P*-value < 0.05: Significant;* P*-value < 0.01: highly significant

## Discussion

Severe MS causes many functional and morphological changes in the left atrium due to chronic resistance to active and passive emptying and a chronic increase in left-atrium pressure. Left-atrial structural and functional remodeling is expected to occur after the relief of mitral obstruction by balloon valvuloplasty [9]. Many studies have investigated the changes after BMV, but most have focused on the immediate or short-term effects using standard 2D Echocardiography, and there is not much data on long-term effects or speckle-tracking measures. This study evaluated the outcomes of BMV either immediately after the procedure and at different time points using 2D speckle-tracking Echocardiography. The results demonstrated that LA GLS, sPAP, MVA, and the LAV index were significantly lower in patients with severe MS compared to the control group. However, all of these parameters improved immediately after BMV and at 6 and 12 months (*p* < 0.01).

This study was extended to compare various distinctive parameters before and after BMV, as detailed in Tables 7, 8, 9. Table 7 presents the BMV and the immediate LA GLS relationship in two patient groups categorized by the MVA's severity. The results indicate that the severity of MVA impacts the immediate LA GLS following BMV. The severe group consists of 44 patients with severe MVA but not as severe as the second group. The more severe group includes 51 patients with an even more severe condition characterized by a smaller MVA. Patients with more severe MVA (MVA less than 1.0 cm2) tend to have a lower immediate LA GLS than those with severe MVA (MVA less than 1.5 cm2). The value of 0.03* suggests a statistically significant difference in immediate LA GLS between these groups.

**Table 7:** The pre-BMV MVA effect on the post-BMV immediate LA GLS

Variable	Severe cases (n = 44) (MVA less than 1.5 cm^2^)	More severe cases (n = 51) (MVA less than 1.0 cm^2^)	*p* value (Sig.)
Immediate left atrial global longitudinal strain	37.3 ± 4.7	35.0 ± 5.2	0.03^**^

T-test was used

**:* P*-value > 0.05: Non-significant;* P*-value < 0.05: Significant;* P*-value < 0.01: highly significant

**Table 8:** Relation between gender and Immediate left atrial global longitudinal strain

Variable	Males (n = 24)	Females (n = 71)	*p* value (Sig.)
Immediate left atrial global longitudinal strain	35.9 ± 4.8	36.1 ± 4.9	0.86^NS^

T-test was used

*NS* Non-Significant

**Table 9:** MR and Immediate LA GLS

Variable	Mild MR cases (n = 79)	Moderate MR cases (n = 9)	Moderate to severe MR cases (n = 7)	*p* value (Sig.)
Immediate LA GLS	37.1^a^ ± 4.8	33.3^b^ ± 4.9	27.8^c^ ± 5.2	< 0.01**

One-way ANOVA and Duncan test were used

^a,b,c^Means with the different superscript letters had significant differences with each other

**:* P*-value > 0.05: Non-significant;* P*-value < 0.05: Significant;* P*-value < 0.01: highly significant

Table 8 examines the potential relationship between gender and immediate LA GLS. The results suggest that the Improvement of LA GLS was higher in females. However, the data indicates that gender does not appear to significantly explain variations in immediate LA GLS values in this study.

Table 9 investigates the relationship between MR severity and the immediate LA GLS. Mild MR cases had a significantly higher immediate LA GLS, followed by moderate MR cases, then severe MR cases. The severity of MR is strongly associated with the immediate LA GLS values. Patients with more severe MR tend to have lower immediate LA GLS values, indicating impaired left atrial function. The statistical significance reinforces the validity of these findings.

Our results are consistent with those of Reddy et al. [5], who performed strain imaging to evaluate the early effects of PBMV on the mechanics of the left atrium for 29 patients with MS. They compared the results with those of 30 age- and sex-matched healthy control subjects. The MS cases showed a significant increase in the mean left atrial diameter and left atrial area compared to the control group. Also, the MS cases had significantly lower left atrial strain at baseline compared to the control group. Similar to our results, they observed that patients with severe MS exhibited impaired LA GLS, which improved within 24–48 h after BMV (*p* < 0.001). Furthermore, they found a significant decrease in the mean mitral gradient (MMG) and sPAP (both *p* < 0.001) and A significant rise IN MVA (*p* < 0.001) after BMV.

Ansari et al. [12] investigated the immediate and late outcomes of BMV in the left atrium and left atrial appendage (LAA) in patients who had severe MS with sinus rhythm. They found no considerable improvement in the fractional LAA area change (LAAAC) directly after percutaneous transvenous mitral commissurotomy (PTMC), but significant improvement was observed by 6 months after PTMC. Also, a considerable increase was observed in LAA PW Doppler velocities (LAALDE, LAAEDE, and LAAF velocity) directly after PTMC, which improved significantly within 6 months of follow-up.

Additionally, a considerable rise in LAA DTI velocities was noted (ALAA, ELAA, and SLAA velocity) directly after PTMC, leading to significant improvement within 6 months of follow-up. The MVA increased significantly after PTMC (*p* < 0.001). In contrast, both left atrial volume indexes and PASP decreased significantly after PTMC (*p* < 0.001). However, that study focused on the appendage function, not the LA GLS, and there was no healthy control group, unlike our study.

Our results agree well with a recent study by Samart et al. [13], who observed significant improvement in the MVA after BMV (*p* < 0.001). Also, a significant improvement (24% compared to baseline) was achieved in peak atrial longitudinal strain immediately after the procedure (*p* < 0.001), and the improvement continued as of 3 months after BMV (74% compared to baseline; *p* < 0.001). The left atrial volume index significantly decreased immediately after the procedure (*p* = 0.003) and at the 3-month follow-up (*p* = 0.002). The left atrium volume and left atrium volume index were notably reduced at 24 h after the procedure and during follow-up. Although their results were similar to our study, the follow-up was only for 3 months, and there was no control group.

Rohani et al. [14] reported an improvement in the peak systolic LA GLS after MVR (*p* = 0.012) and after PTMC (*p* < 0.001). Also, the results showed a significant reduction in the estimated PASP after MVR (*p* = 0.006) and BMV (*p* < 0.001). In addition, MS patients' mean MVA was significantly increased after BMV (*p* < 0.001). Despite having similar results regarding BVM, there was no significant difference in PALS after PTMC and MVR (*p* = 0.60). The results were also a combination of the outcomes of both PTMC and MVR with no assessment of the short and long-term outcomes of only BMV.

In the present study, a significant positive correlation was found between immediate LA GLS and the achieved MVA (0.64, *p* < 0.01), and the function class was improved significantly after valvuloplasty (*p* < 0.01). This correlation was unique to our study and reflected the efficiency and success of BVM in enhancing both the left atrial volume and function. Ahmed [15] found a significant positive correlation between the left atrial longitudinal strain improvement and drop in the mean trans-mitral pressure gradient, as well as left atrial volume reductions at 12 months after BMV. Rohani et al. [14] reported a significant correlation between the drop in mean transmitral pressure gradient and left atrial global longitudinal strain (r = 0.60, *p* < 0.01) after PTMC. However, a non-significant correlation was observed between the PALS and the MVA (r = 0.03). Many studies have also demonstrated the improvement of function class after a successful BMV [6, 13, 15].

This study had some limitations, such as a lack of investigation of BMV's effects on the right and left ventricular strain. Another limitation of this study is that we focused on the reservoir function only, although it contributes 70% of the left atrial function. The reason was that the equipment used at the time of the study was limited to only determining the reservoir function, so further studies are needed to include all functions of the left atrium. However, the study had some strengths, such as a relatively large sample size of case and control groups and a relatively long-term follow-up compared to previous studies.

## Conclusions

This study estimated the immediate, short-term, and long-term outcomes of BMV in patients with severe MS. 95 patients who underwent successful BMV and 80 healthy control subjects were included. The LA GLS has been identified as a potential indicator of left atrial function (mainly its reservoir function). Moreover, improved LA GLS after valvuloplasty could be a promising indicator of positive outcomes following BMV. Further studies are needed to confirm our findings. More research is also warranted to explore the effects of BMV on all functions of the left atrium and to address the study's limitations. Overall, these results show the valuable effects of BMV in improving left atrial function and provide insights for managing severe MS.

## Data Availability

The dataset used during the current study is available from the corresponding author upon reasonable request.

## Abbreviations

BMV: Balloon mitral valvuloplasty
LA: Left atrial
LAA: Left atrial appendage
LAAAC: Left atrial appendage fractional area change
LAAEDE: Left atrial appendage early diastolic emptying velocity
LAAF: Left atrial appendage filling velocity
LAALDE: Left atrial appendage late diastolic emptying velocity
LA GLS: Left atrial global longitudinal strain
LAV: Left atrial volume
MMG: Mitral mean gradient
MS: Mitral stenosis
MVA: Mitral valve area
MVR: Mitral valve replacement
PALS: Peak atrial longitudinal strain
PBMV: Percutaneous balloon mitral valvuloplasty
PTMC: Percutaneous transvenous mitralcommissurotomy
sPAP: Systolic pulmonary artery pressure
